# Diffuse Cavernous Hemangioma of the Rectum Diagnosed after a 15-year History of Recurrent Gastrointestinal Bleeding: A Case Report

**DOI:** 10.1159/crg/abwag015

**Published:** 2026-09-29

**Authors:** Kazuhiko Obata, Kazutake Okada, Kan Uchiyama, Ryuzo Murai

**Affiliations:** a Department of Gastroenterology Onaka-kai Onaka Clinic, Tokyo, Japan; b Department of Surgery, Tokai University School of Medicine, Hachioji, Tokyo, Japan; c Division of Gastroenterology and Hepatology, Department of Internal Medicine, The Jikei University Kashiwa Hospital, Kashiwa, Chiba, Japan

**Keywords:** Diffuse cavernous hemangioma, Hematochezia, Laparoscopic intersphincteric resection, Rectum, Sphincter-preserving surgery

## Abstract

**Introduction:**

Diffuse cavernous hemangioma of the rectum (DCHR) is a rare benign vascular lesion of the gastrointestinal tract that typically manifests in childhood with recurrent painless hematochezia. Owing to its rarity and nonspecific presentation, DCHR is frequently misdiagnosed as hemorrhoidal disease or inflammatory bowel disease, culminating in substantial diagnostic delay. Reports on laparoscopic surgery for DCHR are limited.

**Case Presentation:**

A 26-year-old woman was referred to our institution for the evaluation of microcytic anemia and recurrent painless hematochezia associated with defecation since childhood, persisting for over 15 years. Lower gastrointestinal endoscopy revealed bluish-purple dilated and tortuous vessels throughout the rectum, accompanied by mucosal swelling and congestion. Contrast-enhanced computed tomography (CT) demonstrated diffuse rectal wall thickening with gradual enhancement and serpentine dilated vessels with numerous phleboliths in the mesorectum, leading to a diagnosis of DCHR. Laparoscopic intersphincteric resection (Lap-ISR) with a diverting loop ileostomy was performed, with an estimated blood loss of 310 mL. Histopathological examination revealed numerous diffusely dilated, thin-walled vessels extending from the submucosa to the subserosa, with phleboliths exhibiting calcification. The diverting ileostomy was closed 4 months postoperatively. Eight months after surgery, neither hematochezia nor anemia has recurred, and anorectal function has been well preserved.

**Conclusions:**

Early clinical suspicion is crucial to ensure the timely diagnosis of DCHR in young patients presenting with recurrent painless hematochezia dating from childhood. Lap-ISR represents a feasible sphincter-preserving approach for extensive, transmurally involved DCHR, enabling complete excision while sparing the patient a permanent stoma.

## Plain Language Summary

Diffuse cavernous hemangioma of the rectum is a rare, noncancerous condition in which the small blood vessels in the wall of the lower bowel become enlarged and twisted. The condition usually begins in childhood and causes repeated episodes of painless rectal bleeding. Because these symptoms closely resemble those of hemorrhoids or bowel inflammation, the correct diagnosis is often delayed for many years. A 26-year-old woman was referred to our clinic with long-standing anemia (a low red blood cell count) and painless rectal bleeding persisting for over 15 years. A camera examination of the lower bowel (colonoscopy) and a CT scan – a detailed X-ray examination – showed swollen, bluish blood vessels and thickening of the bowel wall with tiny calcified spots, confirming the diagnosis. She underwent keyhole surgery that removed the diseased rectum while preserving the muscles that control bowel movements, together with a temporary stoma (an opening of the small bowel on the abdominal wall) to protect the surgical site. The stoma was closed 4 months later. Eight months after surgery, she remained free of bleeding and anemia, and her bowel control remained normal. This rare condition should be considered early in young patients with long-lasting painless rectal bleeding beginning in childhood, so that an accurate diagnosis can be reached without delay. Once diagnosed, surgery that preserves the muscles that control bowel movements can completely remove the diseased rectum, avoid the need for a permanent stoma, and maintain the patient’s quality of life.

## Introduction

Diffuse cavernous hemangioma of the rectum (DCHR) is a rare benign vascular disorder of the gastrointestinal tract, with an average age of onset of 6 years [1]. In addition to the rectum, this entity may also involve the small intestine – particularly the jejunum – as well as the liver, spleen, and urinary bladder [2]. The predominant clinical manifestation is painless rectal bleeding, although complications, including intestinal obstruction and bowel perforation, have also been documented [1]. Definitive management consists of surgical resection; however, balancing complete disease eradication with the preservation of anal sphincter function remains a significant clinical challenge. While the utility of standard laparoscopic approaches has previously been reported [3, 4], experience with ultra-low sphincter-preserving techniques is limited. Herein, we present a case of a successful laparoscopic intersphincteric resection (Lap-ISR) in a 26-year-old patient diagnosed with DCHR following a 15-year diagnostic delay, highlighting the feasibility of this approach for complete symptom resolution and functional preservation. This case report was prepared in accordance with the CARE Checklist, and the completed checklist is provided as online supplementary material (for all online suppl. material, see https://doi.org/10.1159/crg/abwag015).

## Case Presentation

A 26-year-old woman was referred to our institution for the assessment of anemia and recurrent painless rectal bleeding during defecation since childhood. She had no significant medical or family history, no known congenital conditions, and reported no allergies or regular medication use. Physical examination revealed no abdominal tenderness. Digital rectal examination revealed circumferential edematous mucosa located approximately 2 cm from the anal verge. Laboratory investigations revealed microcytic anemia (hemoglobin, 6.8 g/dL; hematocrit, 26.7%; mean corpuscular volume, 70 fL) consistent with iron deficiency (serum iron, 33 µg/dL; ferritin, 10.4 ng/mL). Additional laboratory evaluation demonstrated a serum albumin level of 3.9 g/dL, consistent with adequate nutritional status, and an elevated D-dimer level of 4.5 µg/mL, suggestive of microthrombus formation. Colonoscopy revealed tortuous, dilated bluish-purple vessels extending from the rectum above the peritoneal reflection to the rectosigmoid junction (Fig. 1a), including marked mucosal congestion and swelling from the rectum below the peritoneal reflection to just above the dentate line (Fig. 1b).

**Fig. 1.**
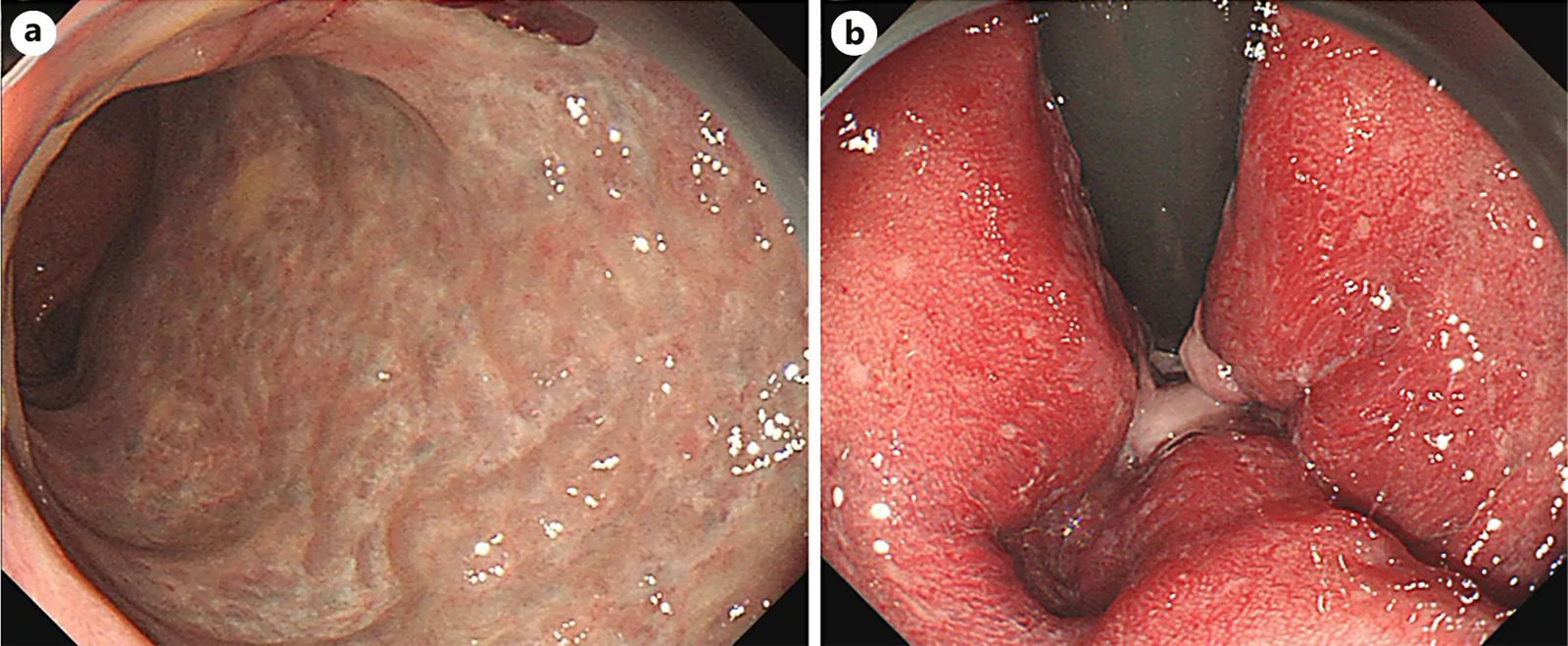
Endoscopic images of the patient. **a** Multiple bluish-purple varicosities representing tortuous vascular dilatation from the rectum above the peritoneal reflection to the rectosigmoid colon. **b** Congestion and swelling of the mucosa from the rectum below the peritoneal reflection to just above the dentate line.

Contrast-enhanced abdominal computed tomography (CT) demonstrated rectal wall thickening with gradual contrast enhancement from the arterial phase (Fig. 2a) through the portal venous phase (Fig. 2b) to the equilibrium phase (Fig. 2c), accompanied by serpentine dilated vessels and numerous phleboliths in the rectal mesentery. Oral iron therapy alone led to little improvement in anemia; therefore, surgery was planned after informed consent was obtained. Preoperative colonoscopy was used to determine the resection margins, which extended from the rectosigmoid colon to just above the dentate line and included the involved segment. After a 3-month preoperative preparation period following diagnosis, Lap-ISR with diverting loop ileostomy was performed. Intraoperatively, the hemangioma involved nearly the entire rectum and extended to the serosal surface (Fig. 3a, b). After confirming that the hemangioma had not invaded the anal sphincter, careful dissection was performed in the intersphincteric plane to preserve anal sphincter function, with partial preservation of the internal sphincter. The rectal mucosa above the dentate line was completely resected transanally, and the proximal resection margin was set at the unaffected sigmoid colon. Hand-sewn coloanal anastomosis and a diverting ileostomy were performed. Intraoperative blood loss was 310 mL, without hemodynamic compromise. The resected specimen measured 125 × 190 mm. Macroscopic examination revealed a dense proliferation of dilated blood vessels on the serosal surface (Fig. 3c) and absence of mucosal folds with multiple small, clustered nodules on the mucosal surface (Fig. 3d).

**Fig. 2.**
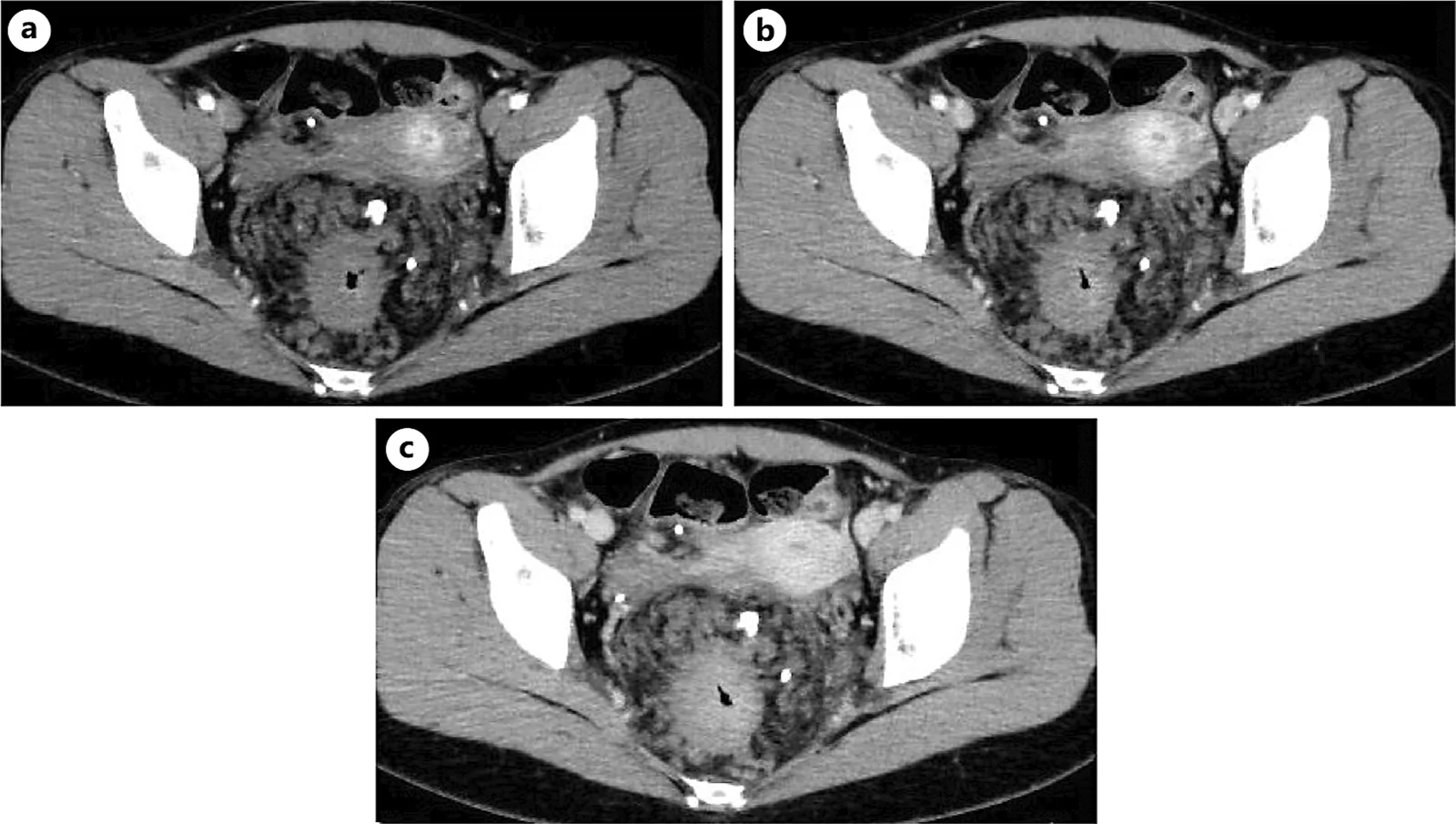
Contrast-enhanced abdominal CT findings. Arterial (**a)**, portal venous (**b**), and equilibrium phases (**c**). CT shows the rectal wall thickening with gradual contrast enhancement and serpentine dilated vessels with numerous phleboliths in the rectal mesentery.

**Fig. 3.**
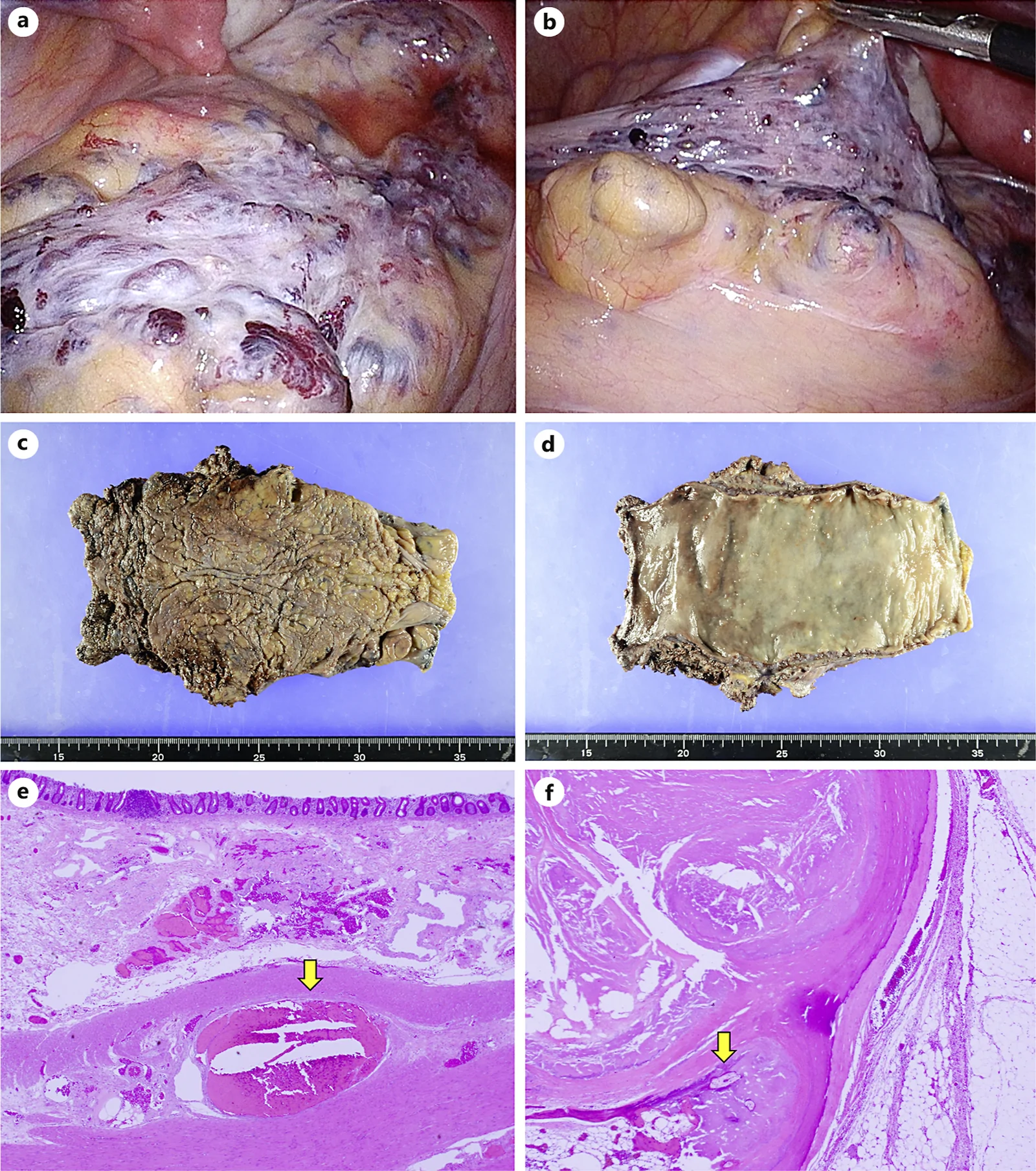
Intraoperative findings. **a** Hemangioma occupying nearly the entire serosal surface of the anterior rectal wall. **b** Confirmed multiple small nodules formed by vascular calcification in the rectal mesentery. Macroscopic findings of the resected specimen – serosal (**c**) and mucosal surfaces (**d**). The serosal surface shows a dense proliferation of dilated blood vessels, whereas the mucosal folds are absent and replaced by multiple small, clustered nodules on the mucosal surface. **e**, **f** Histopathological evaluation of the extracted specimen using hematoxylin and eosin staining. **e** The mucosal surface is relatively preserved, whereas the submucosal to subserosal layers show a diffuse proliferation of variably sized, dilated, thin-walled cavernous vascular spaces. Calcified phleboliths are identified within the dilated veins (arrow). **f** Higher magnification view of a markedly dilated vein in the subserosal layer, demonstrating a phlebolith with concentric lamellar calcification (arrow) surrounded by a thickened fibrous venous wall.

Histopathological examination revealed numerous diffusely dilated thin-walled vessels extending from the submucosa to the subserosa of the rectum (Fig. 3e). Within the markedly dilated veins, multiple phleboliths with concentric lamellar calcification surrounded by thickened fibrous venous walls were identified (Fig. 3f). These findings were consistent with the diagnosis of DCHR. Postoperatively, the patient developed outlet obstruction and a high-output stoma, both of which improved with conservative treatment. The patient was discharged in a stable condition on the 29th postoperative day. The diverting ileostomy was closed 4 months postoperatively. At 8 months after surgery, the patient remained free of hematochezia, with the hemoglobin level recovering to 13 g/dL. Long-term functional outcomes were assessed 8 months postoperatively using the Wexner incontinence score and the low anterior resection syndrome (LARS) score. Scores of 8 and 18, respectively, with the latter indicating no LARS, demonstrated well-preserved anorectal function, no restrictions in activities of daily living, and a patient-reported satisfactory functional outcome. The complete clinical course, spanning from symptom onset at approximately 10 years of age to uneventful recovery, is illustrated in Figure 4.

**Fig. 4.**
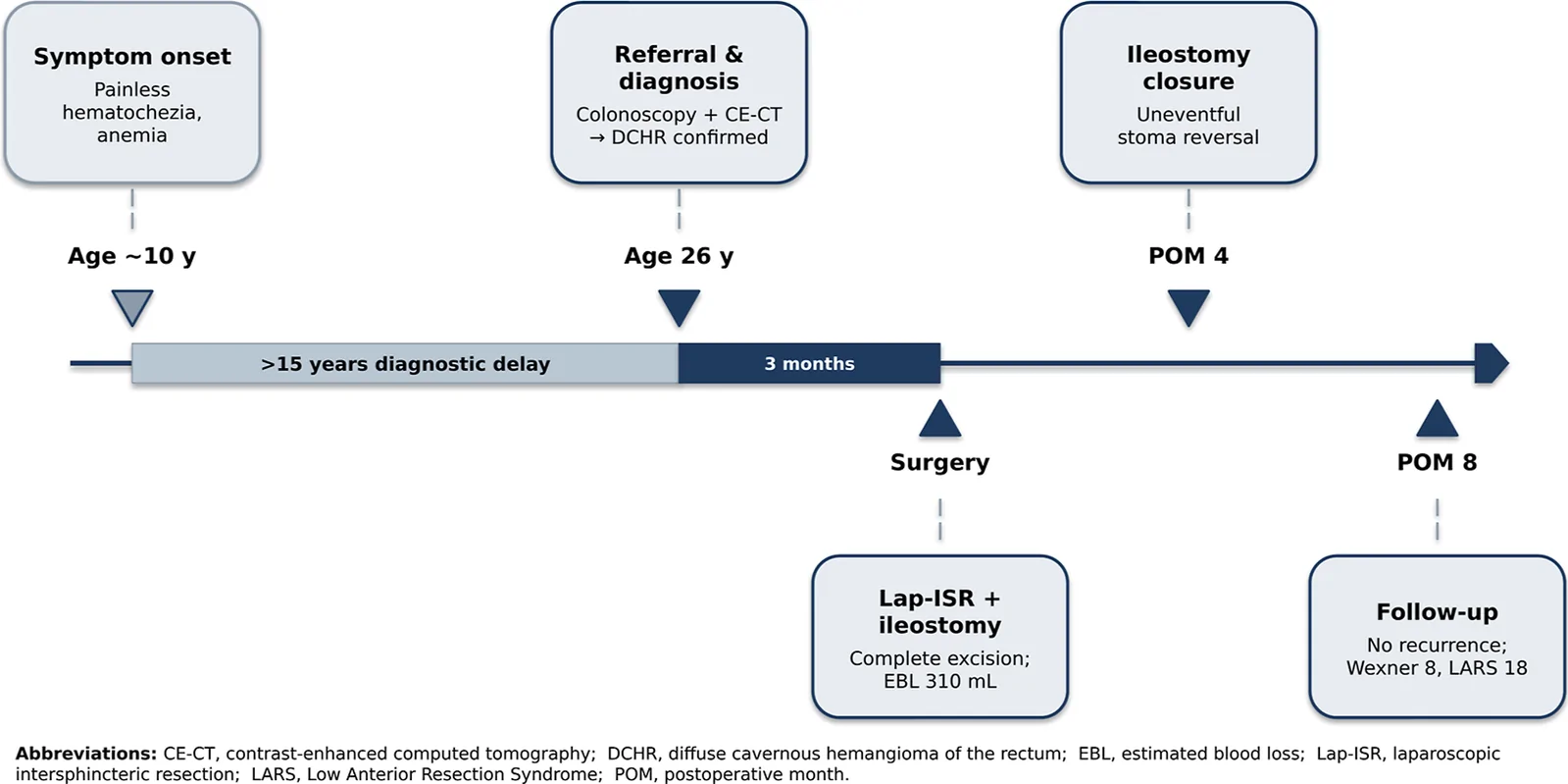
Clinical timeline of the present case. Symptom onset occurred at approximately 10 years of age. A diagnostic delay of more than 15 years elapsed under presumed hemorrhoidal disease before referral and diagnostic work-up at 26 years of age (colonoscopy and CE-CT). Lap-ISR with diverting loop ileostomy was performed 3 months after diagnosis; the ileostomy was closed at POM 4, and the patient achieved sustained clinical remission without recurrence at POM 8. CE-CT, contrast-enhanced computed tomography; DCHR, diffuse cavernous hemangioma of the rectum; EBL, estimated blood loss; Lap-ISR, laparoscopic intersphincteric resection; LARS, low anterior resection syndrome; POM, postoperative month.

## Discussion

According to Pohlen’s classification of gastrointestinal angiomas [5], DCHR is considered a progressive intestinal hamartoma, representing a borderline lesion between vascular malformations and neoplasms. Of note, under the current International Society for the Study of Vascular Anomalies classification [6], lesions historically termed “cavernous hemangioma” are more accurately classified as slow-flow venous malformations based on their non-proliferative endothelium. However, we use the conventional term “DCHR” throughout this report for consistency with the existing literature. Patients typically present from childhood with recurrent, intermittent hematochezia and anemia, and are often misdiagnosed as having hemorrhoids, inflammatory bowel disease, or rectal varices related to portal hypertension [7]. Consequently, the interval from symptom onset to diagnosis averages 17.63 years [1], and such repeated misdiagnoses frequently prompt inappropriate surgical interventions, including hemorrhoidectomy. This is illustrated by Jeffery et al. [8], who reported that 80% of patients with DCHR had undergone at least one unnecessary operation before the correct diagnosis was established. In the present case, symptomatic treatment for presumed hemorrhoids continued for over 15 years before DCHR was correctly identified.

Endoscopically, DCHR may present in two distinct patterns: elevated bluish-purple varicosities and ischemic colitis-like changes with mucosal erosions [1]. The two patterns coexisted in the present case, demarcated by the second Houston valve, and the endoscopic findings in the lower rectum were considered to reflect impaired blood supply. Typical CT findings include rectal wall thickening and scattered calcifications caused by organized thrombi in the surrounding tissue [9]. On magnetic resonance imaging (MRI), the rectal wall typically appears thickened, with low signal intensity on T1-weighted images and high signal intensity on T2-weighted images [3]. Hasegawa et al. [9] reported that fat-suppressed MRI sequences can accurately distinguish hemangiomas from the surrounding rectal mesentery and that endorectal coil MRI – conventionally used to assess the depth of rectal cancer invasion – can also evaluate hemangiomatous infiltration into the anal canal. However, MRI could not be performed in the present case because of claustrophobia. Conversely, angiography is not recommended for routine evaluation because its diagnostic yield is limited by reduced blood flow from multiple thrombi within the hemangioma [1, 7]. In the present case, lower gastrointestinal endoscopy revealed markedly dilated submucosal vessels within the rectum, raising the possibility of an underlying vascular malformation or hemodynamic abnormality. This finding prompted contrast-enhanced CT, which ultimately proved pivotal in establishing the diagnosis.

Non-surgical approaches, including sclerotherapy and transcatheter embolization, have been reported to be insufficiently effective in DCHR, and complete surgical resection remains the standard treatment strategy [3, 10]. As DCHR commonly originates from the dentate line, inadequate resection frequently leads to recurrent rectal bleeding [1]. Accurate delineation of the distal margin of the hemangioma is therefore crucial in determining the optimal surgical strategy. Abdominoperineal resection remained the standard treatment procedure until 1970, despite concerns regarding permanent colostomy and complications, such as bladder dysfunction [11]. Subsequently, the coloanal sleeve anastomosis procedure proposed by Jeffery et al. [8] in 1976 was associated with recurrence due to residual rectal tissue. Although laparoscopy affords improved visualization and facilitates rectal mobilization, reports on laparoscopic surgery for DCHR remain limited. Fu et al. [3] and Leal et al. [4] each described two cases managed by laparoscopic-assisted bowel resection, with uneventful postoperative recovery and complete cessation of rectal bleeding. More recently, both transanal total mesorectal excision and robot-assisted resection have emerged as safe and feasible therapeutic options for DCHR, with minimal intraoperative blood loss reported in both modalities [12, 13]. In this evolving landscape of minimally invasive approaches, the selection of the technique must be tailored to the anatomical extent of the lesion and the patient’s functional considerations. In the present case, the lesion extended to just proximal to the dentate line; therefore, a transabdominal ultra-low anterior resection would have left residual disease on the anal side, as the distal transection line could not have been advanced sufficiently below the lesion. Furthermore, because cavernous hemangiomas characteristically involve the entire rectal wall, from the submucosa to the outermost layer, a coloanal sleeve anastomosis with rectal mucosectomy could have left a residual lesion within the retained muscular cuff, posing a risk of recurrent bleeding. Although abdominoperineal resection could have achieved complete excision, imposing a permanent colostomy on a young woman with a benign disease was deemed clinically unjustifiable, given its profound and lifelong impact on quality of life, body image, and social and reproductive function. Therefore, Lap-ISR was selected to achieve complete excision of the lesion, minimize the risk of recurrence, and preserve anal function. Although the indications for Lap-ISR have historically been limited primarily to ultra-low rectal cancer, our experience suggests that its therapeutic application may extend to extensive, highly vascular benign lesions such as DCHR. To the best of our knowledge, published reports of Lap-ISR for DCHR remain scarce; a PubMed (National Library of Medicine) search using the terms “cavernous hemangioma,” “rectum,” and “laparoscopic intersphincteric resection” identified no prior reports. The present case therefore contributes to the growing literature on sphincter-preserving minimally invasive strategies for DCHR. Given the rarity and heterogeneity of the disease, an individualized surgical strategy is essential, and further large-scale studies are warranted to clarify the safety and utility of these minimally invasive modalities.

This case has several limitations that warrant careful consideration. First, the postoperative follow-up period was limited to 8 months, allowing confirmation of early symptomatic improvement but precluding reliable evaluation of long-term outcomes. DCHR carries an inherent risk of delayed recurrence after incomplete resection, which may become apparent only years after surgery. Second, bowel dysfunction following ISR – including LARS, altered stool frequency, urgency, and passive incontinence – may progress or evolve over time and cannot be adequately assessed within a short-term observation period. Although the patient demonstrated encouraging functional outcomes at 8 months (Wexner score 8, LARS score 18), continued long-term surveillance remains critical to monitor for late recurrence and assess the durability of anorectal function and overall quality of life.

In conclusion, this case underscores the importance of exercising early clinical suspicion for DCHR in young patients with chronic painless hematochezia since childhood so as to prevent prolonged diagnostic delay. Once diagnosed, Lap-ISR offers a viable sphincter-preserving option for extensive DCHR with transmural involvement, achieving complete resection while avoiding a permanent stoma.

## Acknowledgments

We would like to thank Editage (www.editage.com) for English language editing.

## Statement of Ethics

Ethical approval was not required for this case report in accordance with institutional policies. All procedures conformed to the 1964 Declaration of Helsinki and its later amendments. Written informed consent was obtained from the patient for the procedures described and for publication of this case report. We used the CARE reporting guideline [14] to draft this manuscript, and the CARE reporting Checklist [15] when editing, included in online supplement A.

## Conflict of Interest Statement

The authors have no conflicts of interest to declare.

## Funding Sources

This study was not supported by any sponsor or funder.

## Author Contributions

Kazuhiko Obata served as the first and corresponding author. Kazuhiko Obata and Kazutake Okada were responsible for data collection, analysis, and preparation of the initial draft. Kan Uchiyama and Ryuzo Murai critically revised the manuscript. All authors have read and approved the final version of the manuscript.

## Data Availability

All data generated or analyzed during this study are included in this article and its online supplementary material files. Further inquiries can be directed to the corresponding author.
